# Supplementary material for: Preliminary Study: Proteomic Profiling Uncovers Potential Proteins for Biomonitoring Equine Melanocytic Neoplasm
Source: Animals (Basel). 2021 Jun 27;11(7):1913. doi: 10.3390/ani11071913 (PMC8300200; doi:10.3390/ani11071913)
Supplement: Supplementary file 1 [file animals-11-01913-s001.zip › animals-1241036-supplementary.pdf]

Table S1. List of unique proteins from differentially expressed proteins analysis under Wilcoxon rank-sum test and multiple testing via false discovery rate (FDR) correction between normal and mild EMN stages

Protein ID	Protein name	P-value	Adjusted p-value	Normal Horse (N)										Mild EMN Horse (M)					
				N-01	N-02	N-03	N-04	N-05	N-06	N-07	N-08	N-09	N-10	M-01	M-02	M-03	M-04	M-05	M-06
A0A286XW26	Leucine rich glioma inactivated 1 (LGI1)	0.021	0.048	0	0	0	0	0	0	0	0	0	0	15.899	14.811	15.544	0	0	0
A0A5F9DI53	Sodium/potassium-transporting ATPase subunit alpha (Na+/K+-ATPase)	0.005	0.048	0	0	0	0	0	0	0	0	0	0	15.277	15.507	14.661	0	0	14.693
G1NTJ3	WD repeat domain 78 (WDR78)	0.021	0.048	0	0	0	0	0	0	0	0	0	0	14.956	14.519	13.852	0	0	0
G1P1V8	N(alpha)-acetyltransferase 16 (NAA16)	0.021	0.048	0	0	0	0	0	0	0	0	0	0	11.422	16.212	12.405	0	0	0
G1PX76	Storkhead box 1 (STOX1)	0.021	0.048	0	0	0	0	0	0	0	0	0	0	14.334	13.505	15.484	0	0	0
G1Q3S1	Phospholipid phosphatase 6 (PLPP6)	0.005	0.048	0	0	0	0	0	0	0	0	0	0	14.522	12.615	15.449	15.602	0	0
G1QEM5	WD repeat domain 90 (WDR90)	0.021	0.048	0	0	0	0	0	0	0	0	0	0	15.120	14.146	16.354	0	0	0
G1TD38	Centriolar coiled-coil protein 110 (CCP110)	0.021	0.048	0	0	0	0	0	0	0	0	0	0	13.596	12.926	17.573	0	0	0
G1TDZ5	Tumor suppressor candidate 1 (TUSC1)	0.021	0.048	0	0	0	0	0	0	0	0	0	0	18.166	16.543	18.888	0	0	0
H0UXW0	Four and a half LIM domains 5 (FHL5)	0.021	0.048	0	0	0	0	0	0	0	0	0	0	15.491	13.558	0	0	15.136	0
H0V4F2	Plexin D1 (PLXND1)	0.021	0.048	0	0	0	0	0	0	0	0	0	0	14.860	14.946	14.838	0	0	0
H0VKA5	Astrotactin 1 (ASTN1)	0.021	0.048	0	0	0	0	0	0	0	0	0	0	14.963	13.275	9.488	0	0	0
H0W6F7	Dishevelled segment polarity protein 2 (DVL2)	0.021	0.048	0	0	0	0	0	0	0	0	0	0	11.638	12.549	0	14.567	0	0
